# Discrimination of *Malus* Taxa with Different Scent Intensities Using Electronic Nose and Gas Chromatography–Mass Spectrometry

**DOI:** 10.3390/s18103429

**Published:** 2018-10-12

**Authors:** Junjun Fan, Wangxiang Zhang, Ting Zhou, Dandan Zhang, Donglin Zhang, Long Zhang, Guibin Wang, Fuliang Cao

**Affiliations:** 1 College of Forestry, Nanjing Forestry University, Nanjing 210037, China; Junjun.Fan@uga.edu (J.F.); malus2011@163.com (W.Z.); 15905184674@163.com (T.Z.); nanlindandan@126.com (D.Z.); 15298395916@163.com (L.Z.); 13913028679@163.com (F.C.); 2 Co-Innovation Center for Sustainable Forestry in Southern China, Nanjing Forestry University, Nanjing 210037, China; 3 Department of Horticulture, University of Georgia, Athens, GA 30602, USA; Donglin@uga.edu; 4 Yangzhou Crabapple Horticulture Limited Company, Yangzhou 225200, China

**Keywords:** flower scent, intensity, electronic nose, GC–MS, PLSR, crabapple

## Abstract

Floral scent is important in plant reproduction and also has aesthetic implications. However, the accurate determination of aroma is presently limited by the available collection and analysis tools. In this study, the floral scents of four crabapple taxa exhibiting faint, weak, clear, and strong scent intensities were comparatively analyzed by electronic nose (E-nose) and gas chromatography–mass spectrometry (GC–MS). The E-nose was able to effectively group the different taxa in the principal component analysis in correspondence with scent intensity. GC–MS analysis identified a total of 60 volatile compounds. The content of nitrogen-containing compounds and aliphatics and the number of unique components of the more aromatic taxa was significantly higher than the less aromatic taxa. α-Cedrene, β-cedrene, 5-methyl-1,3-dihydro-2H-benzimidazol-2-one, benzyl alcohol, linalool, and 4-pyrrolidinopyridine contributed significantly to taxon separation. The pattern recognition results confirmed that the E-nose results corroborated the GC–MS results. Furthermore, partial least squares regression analysis between the aromatic constituents and sensors indicated that particular sensors were highly sensitive to N-containing compounds, aliphatics, and terpenes. In conclusion, the E-nose is capable of discriminating crabapple taxa of different scent intensities in both a qualitative and quantitative respect, presenting a rapid and accurate reference approach for future applications.

## 1. Introduction

Floral scent plays an important role in the reproductive processes of many plants, as well as in guaranteeing the yield and quality of many economically valuable plants. It also enhances the aesthetic properties of ornamental plants and cut flowers. Traditional scent analysis in plants has typically relied on sensory evaluation methods that perceive types and intensity of odors. Odor intensity is very complex, due to the effect of the odor detection threshold (ODT). ODT is the lowest concentration of a certain odor compound that is perceivable by the human olfactory system. The threshold of a chemical compound may change due to its shape, polarity, partial charges, or the addition of other compounds [1]. Therefore, sensory evaluation has various limitations, including strong subjectivity and poor repeatability.

Chromatographic techniques, such as gas chromatography–mass spectrometry (GC–MS), solid-phase micro extraction (SPME), and headspace analysis, have increasingly been used to identify and quantify the aromatic components of fragrant plants, such as *Silene latifolia* [2], rose [3], *Luculia pinceana* [4], and *Osmanthus fragrans* [5]. However, these methods often fail to provide a global fingerprint of the scent sample, as the compounds detected are typically dependent on the selected sample pretreatment method. Furthermore, they are hampered by their complex technology, high running costs, and prolonged analysis time. In response to chromatographic technologies, an electronic nose (E-nose) has emerged as an olfactory simulation test tool that allows for the high-throughput analysis of volatile organic compounds in a complex matrix [6]. Using specific nano sensor arrays which reflect the changes in conductivity produced by the adsorption of compounds together with pattern recognition software, a global fingerprint of the volatile components in a sample can be obtained. In contrast to chromatographic techniques, which focus on the separation and detection of individual chemical components, the E-nose relies on response and recognition technology, which can rapidly identify and separate complex odors [6]. In addition, the E-nose is able to accurately distinguish the odor of complex samples at a low cost without the need to quantitatively analyze each individual component in the test sample, as is required for GC–MS. Thus far, the E-nose has primarily been used in food processing [7], evaluating the shelf-life of fruits, vegetables [8,9], meat, and aquatic products [10,11], and determining the authenticity of tobacco, alcohol, and other beverages [12,13]. Scholars have recently attempted to train the E-nose to predict the response of the human sensory system to particular odors. For instance, the E-nose was trained to predict odor pleasantness [14] and was found to correlate well with the human data (above the 0.60 level) for single-component odorants [15]. However, none of the models in the pattern recognition software are able to accurately predict the human values or pleasantness for more than a few descriptors. Hence, the prediction of human sensory ratings from instrumental measurements is still arguably the greatest challenge of sensor-based machine olfaction. With regard to floral scent detection, the E-nose has been applied to germplasm differentiation [16,17], flowering stage distinction [18], and flower organ differentiation [19]. If the E-nose can be applied to the fast screening of the interested taxon, such as a strong scent taxon or a special floral scent type, and accurately mapped to sensory evaluation, it would have very important applications in the evaluation of floral scent.

E-nose technology has not been previously applied to the evaluation of scent intensity in crabapple. In this study, using headspace solid-phase micro extraction coupled with gas chromatography–mass spectrometry (HS–SPME)-GC–MS in conjunction with the E-nose, we evaluated the scent characteristics of crabapple (*Malus*, Rosaceae) taxa of different scent intensities. The aims of the study were as follows: (1) to evaluate the main differences in compounds among the different taxa; (2) to assess the ability of the E-nose to distinguish the different taxa; and (3) to explore the relationship between the detected compounds and the different E-nose sensors in order to assess the potential functionality of the E-nose in floral fingerprinting, scent type classification, and scented flower breeding.

## 2. Materials and Methods

### 2.1. Plant Material

More than one hundred *Malus* taxa scent intensities are evaluated by 30 trained assessors (unpublished) using a 6-point scale method [20]. Based on the sensory evaluation results, four taxa with higher ornamental value, *M*. ‘Hillieri’, *M. sylvestris*, *M*. ‘Van Eseltine’, and *M.* ‘Brandywine’, had different scent intensities, categorized as faint, weak, clear, and strong, respectively.

Fresh early-flowering inflorescences of these four *Malus* taxa were collected from the National Crabapple Germplasm Genetic Database (Yangzhou City, Jiangsu Province, China). Each taxon was represented by three different plants. The plants were situated more than 50 m apart, and 10 inflorescences per plant were randomly selected for analysis. Inflorescences from the different taxa were separately placed into deionized water before being transported to the Nanjing Forestry University (Nanjing, China), where they were maintained at room temperature (25 ± 1 °C).

### 2.2. Floral Scent Determination

On the following day, the experiments were carried out at 8:00 a.m.–11:00 a.m. Approximately 4 g of fully expanded flowers were placed into a 200 mL capped SPME vial. After approximately 30 min equilibration between the flower and the headspace, the floral scent was analyzed using the E-nose and HS–SPME-GC–MS. Three replicates were tested for each taxon (4 g flowers from 10 inflorescences per replicate).

#### 2.2.1. E-Nose Analysis

A PEN3 portable E-nose (Airsense Company, Schwerin, German) was used in the experiment. The basic structure of this device consists of a sensor array unit, a sampling apparatus, and pattern-recognition software (Win Muster v.1.6). The sensor array includes 10 metal oxide semiconductor (MOS) sensors, the characteristics of which are indicated in Table 1 [21,22]. The sensor response is reflected as resistivity (Ohm) and relies on the changes in conductivity produced by the adsorption of chemical molecules in the gas state and on the subsequent surface chemical reaction or physical effect.

During the measurement process, the headspace gas was pumped into the sensor chamber at a constant rate of 150 mL·min^−1^. The measurement phase lasted 50 s, which is sufficient to reach a stable state. The interval time was 10 s. In this study, the stabilized response sensor values were selected at 46–48 s to analyze the pattern recognition. To return the sensors to the baseline, a 300 s cleaning phase was undertaken after each measurement. Three replicates were tested for each taxon.

#### 2.2.2. HS-SPME-GC-MS Analysis

HS-SPME extraction was performed using a 65 μm polydimethylsiloxane/divinylbenzene (PDMS/DVB) SPME filed portable sampler(Supelco, Bellefonte, PA, USA), which could be capable of retaining volatile compounds for up to two weeks without significant loss [23]. The fiber was exposed to the headspace of the capped vial to absorb volatile compounds for 0.5 h at room temperature (25 ± 1 °C). Following volatile component absorption, the needle of the SPME was inserted into the GC. In addition, an empty capped vial was used as a blank control, and the samples were injected into the GC in a random fashion. All scent of the samples was extracted at the same time by 12 SPME filed portable samplers, then stored in dry ice, and measured in one day.

The GC system (Thermo Fisher Scientific, Waltham, MA, USA) was equipped with a DB-5MS fused silica capillary column (5% phenylmethyl siloxane, 30 m × 0.25 mm i.d.; 0.25 μm film thickness; Agilent Technologies, Santa Clara, CA, USA). Following HS-SPME extraction, the fiber that had been exposed to the headspace was inserted into the GC injector port for desorption at 250 °C for 5 min in splitless mode. Helium was used as the carrier gas at a constant flow rate of 1.0 mL·min^−1^. The column oven temperature program was as follows: 50 °C for 1 min, increasing thereafter at 4 °C·min^−1^ to 120 °C and then held for 1 min, followed by an increase at 1.5 °C·min^−1^ to 140 °C, and then a final increase at 12 °C·min^−1^ to 230 °C, with no hold. The temperature of the transfer line and ion source were 230 and 210 °C, respectively. The electron ionization potential of the mass detector was 70 eV and the scan range was from 35 to 450 amu. Linear retention indices (LRI) of the volatile compounds were calculated using an alkane series standard (C5–C30) (Sigma, St. Louis, MO, USA) under the same conditions. Identification of volatile compounds was made by comparing the mass spectra with the National Institute of Standards and Technology (NIST) 12 library (similarity > 75%) and previous reports on linear retention indices, as well as published index data (SuperScent: http://bioinf-applied.charite.de/superscent/index.php?site=scentsearch; PubChem: http://pubchem.ncbi.nlm.nih.gov/; ScentBase: http://www2.dpes.gu.se/SCENTbase.html). Therefore, in our study, no standard was used, and that the identifications are tentative, based only on MS similarity and LRI. Each taxon sample has three replicates, and mean values with relative standard deviations (mean standard deviation, %) were reported. The relative contents of each volatile constituent were calculated by normalizing the peak area (Xcalibur 3.1 (Thermo Fisher Scientific, Waltham, MA, USA)).

### 2.3. Data Analysis

A biplot, which is the combination of a score and loading plot, was used to illustrate the principal component analysis (PCA) and partial least squares regression (PLSR) results. The non-supervised PCA was used to reveal the distribution of the samples and determine the factors that contributed most to the data separation. This method was used to evaluate the separation of the different taxa based on the E-nose sensors, as well as the contribution of the compounds to the observed data separation. PLSR was used to assess the correlation among the different taxa, E-nose sensors, and volatile compounds. The jack-knife method was used to assess the significance of the variables. PCA, PLSR, and jack-knife significance testing were performed in The Unscrambler software v. 10.4 (CAMO, Oslo, Norway; http://www.camo.com/). The metabolite variance between the different taxa was analyzed using SPSS v. 19.0 (IBM Corp., Armonk, NY, USA).

## 3. Results

### 3.1. Discrimination of the Different Taxa Using the E-Nose

**Figure 1:**
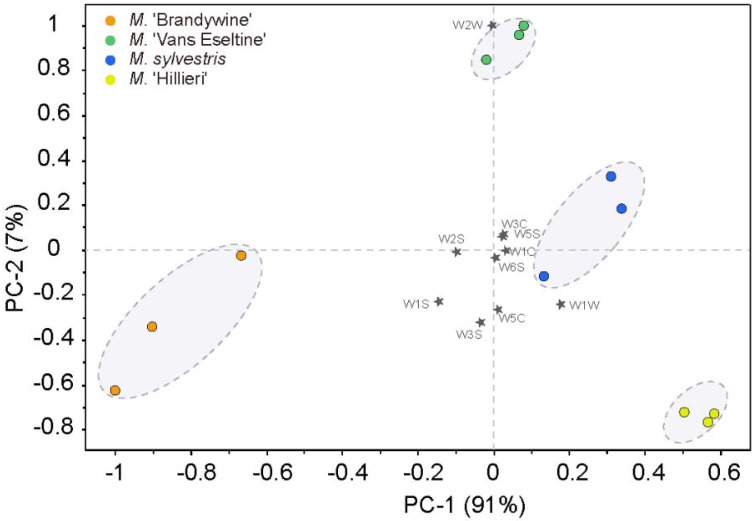
PCA biplot based on the E-nose data of the flowers of the four *Malus* taxa.

PCA was used to evaluate the separation of the different taxa by the E-nose (Figure 1). The first two principal components (PCs) accounted for 98% of the total variance. The four taxa were clearly separated in the plot and were located in both the positive and negative axes of PC1 (91%). The scent intensity showed an increasing trend in a negative direction along the *x*-axis.

The contribution of the sensors to the PCA discrimination was assessed (Figure 1). Sensors W1W, W1S, and W2S influenced PC1 more heavily, whereas sensors W2W, W3S, and W5C made a significant contribution to PC2, as indicated by their longer projections on the axes. The sensor W1W is sensitive to terpenes and sulfurous organic compounds, W1S is sensitive to broad-range methane, and W2S is sensitive to alcohols. Sensors W2W, W3S, and W5C are sensitive to sulfurous organic compounds, methane, and nitrogen oxides, respectively (Table 1). This indicated that terpenes, S-containing compounds, aliphatics, N-containing compounds, and alcohols were probably responsible for the observed separation.

### 3.2. Discrimination of the Different Taxa Using GC–MS

#### 3.2.1. Identification and Comparison of the Volatile Compounds among the Different Taxa

**Figure 2:**
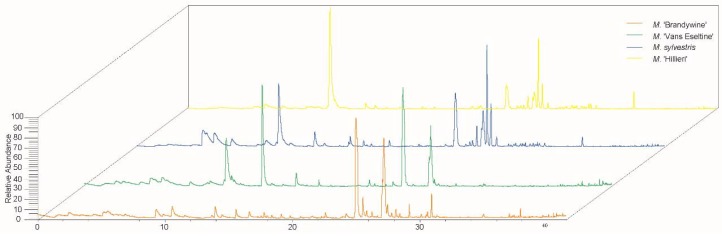
Total ionic chromatogram of the volatile compounds emitted from the flowers of the four *Malus* taxa.

To determine the significant volatile components, the identified aromatic compounds and their relative contents (%) were summarized (Figure 2, Table 2). A total of 60 volatile compounds were putatively identified in the four taxa. There were significant differences in the types and relative contents of volatile compounds in the flowers from the different taxa. The main volatile components in *M*. ‘Brandywine’ was 5-methyl-1,3-dihydro-2H-benzimidazol-2-one (constituting 23.8% of the total content). The primary volatile components in *M*. ‘Van Eseltine’ also included 5-methyl-1,3-dihydro-2H-benzimidazol-2-one, as well as linalool and benzyl alcohol (constituting 50.1% of the total). Benzyl alcohol also constituted the primary volatile compounds in in *M. sylvestris,* along with α-cedrene and 4-pyrrolidinopyridine, constituting 46.3% of the total. Similarly, in *M.* ‘Hillieri’, the main volatiles were benzyl alcohol and α-cedrene (51.7% of the total content).

**Figure 3:**
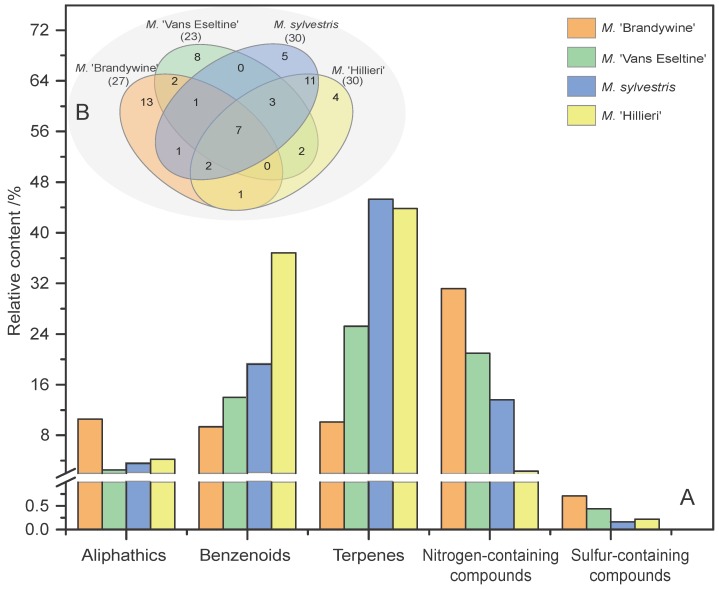
Statistical analysis of the volatile compounds present in the flowers of the four *Malus* taxa. (**A**) Compounds of different chemical classes among the four taxa. (**B**) Venn diagram indicating the similarities and differences in total volatile compounds among the different taxa. The numbers in related overlapping areas indicate the compounds shared between the different taxa.

The relative contents of the different chemical classes (aliphatics, benzenoids, terpenes, N-containing compounds, and S-containing compounds) among the four taxa were calculated and compared, and the results are shown in Figure 3. Interestingly, Terpenes, benzenoids and N-containing compounds were the highest in all four taxa. Among the most aromatic taxa (‘Brandywine’ and ‘Van Eseltine’), the content of N-containing compounds and aliphatics was significantly higher than in the other less aromatic taxa (Figure 3A). The volatile composition of *M. sylvestris* and *M.* ‘Hillieri’ was similar, and these two taxa shared 23 compounds in common and also exhibited a greater diversity of compounds than ‘Brandywine’ and ‘Van Eseltine’ (Figure 3B). Seven compounds were shared between the four taxa (dodecane, linalool, α-cedrene, β-cedrene, geranylacetone, 4-pyrrolidinopyridine, and cocarboxylase). ‘Brandywine’, which is the most aromatic taxon, possessed 13 unique compounds, which was higher than the other taxa (Figure 3B).

#### 3.2.2. PCA Based on the GC–MS Data

**Figure 4:**
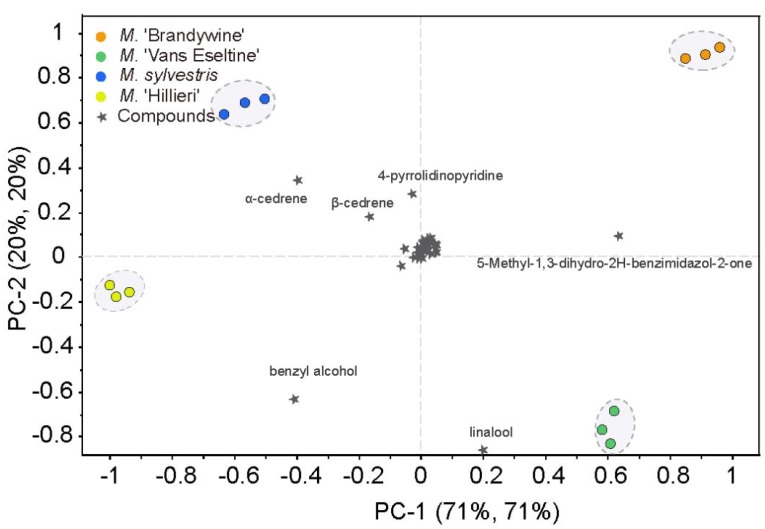
PCA biplot based on the scent compounds of the flowers of the four *Malus* taxa.

To determine the volatile compounds that play a critical role in differentiating scent intensity, the 60 compounds identified using SPME–GC–MS were subjected to PCA. The four taxa could be clearly discriminated based on the first two PCs, which explained 91% of the total variance (Figure 4). Scent intensity increased in a positive direction along the *x*-axis. Several compounds were found to contribute significantly to the discrimination between the four taxa. 5-methyl-1,3-dihydro-2H-benzimidazol-2-one, benzyl alcohol, and α-cedrene contributed greatly to PC1, whereas linalool, benzyl alcohol, 4-pyrrolidinopyridine, α-cedrene, and β-cedrene showed a high contribution to PC2. These six compounds were predominantly N-containing compounds, terpenes, and alcohols, which corroborates our results obtained in Section 3.1, above. The relative contents of these six compounds were high, accounting for 33% to 48% of the total content. These findings allowed for an evaluation of the correlation between the effective volatile compounds and the sensors.

### 3.3. Correlation between E-Nose and GC–MS

**Figure 5:**
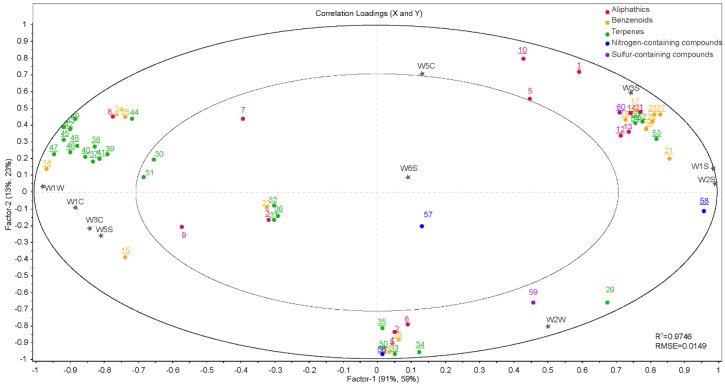
PLSR correlation loadings plot of the sensory attributes, E-nose sensors, and selected compounds of the four *Malus* taxa. The numbers 1–60 correspond to the compound codes indicated in Table 2.

PLSR was used to compare the E-nose measurements and volatile compounds detected by GC–MS. The regression coefficients obtained from the jack-knife significance testing indicated that some of the X-variables (compounds) were significantly correlated (*p* ≤ 0.05) with one or more of the 10 sensors (Figure 5). In PC1, aliphatics, N-containing compounds, S-containing compounds, and sensors W5C, W1S, W2S, W2W, and W3S were all located in the right section of the plot and explained between 50% and 100% of the cross-validated variance, indicating that these variables were significantly positively correlated (*p* ≤ 0.05).

Similarly, terpenes were significantly correlated with sensors W1C, W3C, W5S, and W1W (*p* ≤ 0.05). In addition, benzenoids (alcohols) were also positively associated with sensors W5C, W1S, W2S, W2W, W3S and sensors W1C, W3C, W5S, W1W (*p* ≤ 0.05). However, most benzenoid alcohols are concentrated on the left-hand side of the plot, particularly near sensor W2S (which is sensitive to alcohol), while other benzenoid alcohols are distributed on the right-hand side of the plot.

## 4. Discussion

### 4.1. Correlation between the Scent Discrimination of the E-Nose and Sensory Evaluation

The human olfactory system is highly nonlinear in many respects [38,39], and cross-adaptation, masking, and other processes involved in the human perception of odors further complicate the signal processing in olfaction [40,41]. In addition, sensory evaluation is subjective and depends on the long-term accumulation of human practices and behavior, and thus data standardization is challenging. This study found that the PCA based on the E-nose data and sensory aromatic intensities of the taxa did not exhibit a simple linear correspondence (Figure 1). Furthermore, the contribution of the sensors W1S, W2S, and W1W, which are sensitive to broad range methane, alcohols, and terpenes as well as sulfur organic compounds, respectively, was higher. E-nose recognition is based on the multi-dimensional response values of the sensor to the aromatic components, thereby obtaining an overall fingerprint of the volatile components in a sample rather than the qualitative and quantitative results of one or more components [6]. Therefore, the intensity of the floral aroma recognized by the E-nose considers both the compound and its concentration concurrently.

### 4.2. Correlation between the E-Nose and GC–MS Analysis

As GC–MS is widely used in volatiles analysis, it is thus necessary to evaluate the correlation between the E-nose and the GC–MS results. A comparative evaluation of E-nose and GC–MS allowed for an assessment of the contribution of the compounds detected by the E-nose. Different types of E-nose instruments and sensors have various sensitivities to each component [6]. Data were collected by a particular E-nose, and a prediction model was then established to provide a basis for future research on the same material. In this study, we discovered that specific sensors were highly correlated with majority of compounds in crabapple, thereby producing different sensor behaviors and leading to successful scent type differentiation. For example, N-containing compounds, terpenes, and S-containing compounds were highly associated with sensors W5C, W1W, W2S, respectively. This provided a reference for the establishment of the rapid detection of crabapple flower fragrance in the future.

### 4.3. The Contribution of Compounds to Flower Aroma of Crabapple

The sensory characteristics of floral scent are not only related to the aromatic components and their proportions, but also to the aromatic threshold. The odor detection threshold (ODT) refers to the minimum concentration of a certain volatile compound that is perceivable by the human olfactory system and is the quantitative representation of the intensity of a fragrance [2]. The smaller the threshold, the stronger the aromatic intensity. The threshold for monomeric aromatic substances may change due to the addition of other aromatic substances, i.e., different components may exhibit mutual masking or coordinated enhancement [42]. Studies have shown that a mixture of components with a low ODT has coordinated enhancement, while a mixture with a high ODT will have masking properties [42]. In terms of the aromatic substances detected in this study, the ODTs of 42 of the compounds were found in the literature [24,25,26,27,28,29,30,31,32,33,34,35,36], 32 of which had an ODT less than 1 ppm. Among the six compounds that contributed most highly to the aromatic properties of the taxa, the aromatic thresholds of 5-methyl-1,3-dihydro-2H-benzimidazol-2-one and 4-pyrrolidinopyridine are unknown; the threshold of benzyl alcohol is 5; while the other three compounds (α-cedrene, β-cedrene, and linalool) exhibit extremely low thresholds (ODT < 1 ppm), indicating that these four compounds contribute greatly to the aromatic intensity. Although *M*. ‘Brandywine’ did not have the greatest number of compounds, this taxon did possess the highest numbers of compounds with low ODTs (i.e., high aromatic intensities), while *M.* ‘Hillieri’ possessed the least. These findings explain the greater aromatic intensity of *M.* ‘Brandywine’. Typically, ODT is not included in PCA analyses; however, if the ODT values of all the compounds were measured and combined with the compound detection data in the pattern recognition process, the accuracy of the classification results would be further improved. In addition, there were no obvious differences between the compounds detected in this study and other related studies on apples and crabapples [39,43,44,45,46]. However, the main compounds, constituting the primary compounds detected in these studies, were not completely identical. This phenomenon can primarily be attributed to different sample pretreatment methods. Soxhlet extraction, steam distillation, simultaneous distillation–extraction (SDE), and supercritical fluid extraction (SFE) can be used for fragrance analysis; however, these methods can influence the results and also require many reagents. SPME, dynamic-headspace sampling (DHS), and purge and trap (P&T) can also affect the results due to the selective adsorption of compounds by extraction coating. No standard method for determining floral fragrance thus exists.

## 5. Conclusions

This study is the first to evaluate the volatile constituents of crabapple flowers using E-nose technology. The results indicated that among the more aromatic taxa, the contents of N-containing compounds and aliphatics were significantly higher than the less aromatic taxa. Furthermore, the most aromatic taxon *M*. ‘Brandywine’ possessed a significantly higher number of unique compounds than the other taxa. α-Cedrene, β-cedrene, 5-methyl-1,3-dihydro-2H-benzimidazol-2-one, benzyl alcohol, linalool, and 4-pyrrolidinopyridine were found to contribute greatly to the separation of the different taxa. The E-nose was capable of identifying the different crabapple taxa based on their sensory characteristics, and the sensors W1W, W1S, W2S, W2W, W3S, and W5C played an important role in distinguishing the taxa. The correlation between the aromatic constituents and sensors indicated that particular sensors were more sensitive to N-containing compounds, aliphatics, and terpenes. Based on the results obtained in this study, volatile profiling by GC–MS and E-nose in combination with multivariate statistical analysis constitutes a promising tool for an overall quality evaluation of crabapple flower scent.

## Figures and Tables

**Table 1:** Sensors used in this study and their main application in PEN3.

Sensor No.	Sensor Name	Sensitive Components	Reference, mL·m^−3^ (ppm)
1	W1C	Aromatic compounds	Toluene, 10
2	W5C	Broad-range sensitivity, reacts with nitrogen oxides, very sensitive with negative signal	NO_2_, 1
3	W3C	Ammonia, used as a sensor for aromatic compounds	Benzene, 10
4	W6S	Mainly hydrogen, selectively (breath gases)	H_2_, 100
5	W5S	Alkenes, aromatic compounds, less polar compounds	Propane, 1
6	W1S	Sensitive to methane, broad range, similar to No. 8	CH_3_, 100
7	W1W	Reacts with sulfur compounds, sensitive to many terpenes and sulfur organic compounds, which are important for smell, limonene and pyridine	H_2_S, 1
8	W2S	Detects alcohols, partially aromatic compounds, broad range	CO, 100
9	W2W	Aromatic compounds, sulfur organic compounds	H_2_S, 1
10	W3S	Reacts at high concentrations, sometime very selective (methane)	CH_3_, 10

**Table 2:** Volatile compounds identified in the flowers of four *Malus* taxa using SPME-GC-MS.

Peak	RT	Compound Name	Published ODT/ppm	LRI(calc)	LRI(lit)	Relative Content/%			
						Aroma Intensity: Strong → Faint			
						*M.* ‘Brandywine’	*M*. ‘Vans Eseltine’	*M. sylvestris*	*M*. ‘Hillieri’
		**Aliphathics**							
1	9.3	Methylheptenone	260 [24]	881	964	2.25 ± 0.18Aa	--	--	1.00 ± 0.26Aa
2	9.69	Butyl butanoate	0.1 [25]	906		--	0.39 ± 0.08		--
3	10.12	(Z)-3-Hexenyl acetate	0.0121 [24]	933	1016	--	--	0.48 ± 0.10	--
4	11	(E)-2-Decenal	0.15–5.5 [24]	944	1039	--	0.56 ± 0.07	--	--
5	17.81	(Z)-3-Hexenyl Butyrate	6.8 [26]	1044	1273	0.97 ± 0.18Aa	--	1.17 ± 0.17Aa	--
6	18.41	Dodecane	0.11 [1]	1148	1270	0.46 ± 0.10Bb	1.18 ± 0.19Aa	0.48 ± 0.16Bb	0.60 ± 0.10Bb
7	19.81	(Z)-3-Hexenyl-α-methylbutyrate	0.004 [27]	1121	1203	0.4 ± 0.11Bb	--	1.04 ± 0.20Aa	0.54 ± 0.06Bb
8	22.29	1-Methylnaphthalene	1.4 [24]	1157	*				0.86 ± 0.09
9	22.82	Tridecane	2.14 [28]	1251	1293	--	0.41 ± 0.08Aa	--	0.52 ± 0.02Aa
10	25.86	Texanol	na	1248	*	0.94 ± 0.17Aa	--	0.41 ± 0.08Cc	0.69 ± 0.10Bb
11	26.24	(Z)-3-hexenyl hexanoate	0.0052 [27]	1254	1233	0.88 ± 0.13	--	--	--
12	30.92	2-Tridecanone	0.5 [24]	1344	1496	3.37 ± 0.96	--	--	--
13	37.88	2-Pentadecanone	na	1518	1693	1.06 ± 0.26	--	--	--
14	41.21	Methyl hexadecanoate	4000 [24]	1702	1909	0.22 ± 0.06	--	--	--
		**Benzenoids**							
15	6.09	Styrene	0.12 [23]	676	679	--	1.75 ± 0.11Bb	3.33 ± 0.6Aa	1.65 ± 0.04Bb
16	8.35	Benzaldehyde	0.5 [23]	748	782	--	1.15 ± 0.18	--	--
17	10.55	4-Methylanisole	0.0029 [24]	961	1001	2.18 ± 0.53	--	--	--
18	11.14	Benzyl alcohol	5.5 [24]	925	1030	--	10.45 ± 0.35Cc	13.52 ± 1.45Bb	32.57 ± 0.79Aa
19	13.68	Methyl benzoate	0.028 [24]	1160	1107	0.51 ± 0.24	--	--	--
20	14.48	2-Phenylethanol	0.045 [24]	1211	1129	0.54 ± 0.10	--	--	--
21	15.59	Benzyl nitrile	1–10 [24]	1282	1098	1.87 ± 0.33Aa	0.36 ± 0.06Bb	--	--
22	16.78	Benzyl acetate	<0.001 [27]	1048	1107	--	--	1.88 ± 0.43	--
23	22.62	(2-Nitroethyl)benzene	0.002 [29]	1729	*	0.89 ± 0.08	--	--	--
24	22.95	Cinnamyl alcohol	2.8 [30]	1215	1304	--	--	--	0.97 ± 0.18
25	25.56	4-Methoxyphenethyl alcohol	na	1286	1250	3.35 ± 0.20	--	--	
26	31.29	Cuparene	na	1446	1502	--	--	--	0.48 ± 0.11
27	31.62	2,6-di-*tert*-butyl-4-methylphenol	1 [31]	1450	*	--	--	0.53 ± 0.15Bb	1.17 ± 0.15Aa
28	39.11	Benzyl benzoate	1–10 [24]	1461	1789	--	0.3 ± 0.05	--	--
		**Monoterpenes**							
29	5.16	leaf alcohol	0.01–0.2 [24]	465	552	1.25 ± 0.19Bb	1.86 ± 0.08Aa	0.68 ± 0.15Cc	
30	7.46	α-Pinene	0.12–1.01 [24]	892	943	--	--	2.22 ± 0.67Aa	1.23 ± 0.11Bb
31	10.19	α-Ocimene	na	932	1044	--	--	0.76 ± 0.10	--
32	10.96	Limonene	0.5–0.7 [24]	943	994	--	--	0.44 ± 0.14	--
33	11.81	(E)-α-Ocimene	0.034 [32]	956	1058	--	0.48 ± 0.05	--	--
34	13.94	Linalool	0.0015 [24]	987	1098	2.3 ± 0.32Cc	19.94 ± 0.98Aa	3.04 ± 0.64Bb	1.49 ± 0.10Dd
35	19.38	Limonene oxide	0.01 [33]	1068	1057	--	0.25 ± 0.08	--	--
36	22.15	Bornyl acetate	0.075 [34]	1199	1270	--	--	0.38 ± 0.11	--
		**Sequiterpenes**							
37	26.71	β-Elemen	na	1396	1336	--	--	3.26 ± 0.06Aa	2.73 ± 0.43Aa
38	27.49	α-Cedrene	0.00003–0.00213	1404	1411	1.76 ± 0.13Bb	0.68 ± 0.07Cc	19.51 ± 0.65Aa	19.13 ± 0.7Aa
39	27.82	β-Cedrene	0.00003–0.00213	1408	1418	0.69 ± 0.10Cc	0.34 ± 0.06Dd	7.99 ± 0.65Aa	6.61 ± 0.52Bb
40	28.26	(Z)-Thujopsene	na	1413	1434	--	--	1.52 ± 0.15Aa	1.37 ± 0.28Aa
41	29.75	(+)-α-Longipinene	na	1429	1352	--	--	0.47 ± 0.12Aa	0.44 ± 0.17Aa
42	30.16	ç-Muurolene	na	1434	1476	--	--	0.55 ± 0.14Ab	0.88 ± 0.10Aa
43	30.32	α-Muurolene	na	1435	1491	--	--	0.41 ± 0.10Ab	0.87 ± 0.20Aa
44	30.41	Curcumene	na	1436	1346	--	--	--	0.31 ± 0.10
45	30.53	β-Selinene	na	1438	1521	--	--	0.47 ± 0.12Aa	0.69 ± 0.11Aa
46	30.9	γ-Gurjunene	na	1442	1409	--		0.43 ± 0.14Aa	0.59 ± 0.07Aa
47	31.47	α-Farnesene	2 [24]	1448	1505	--	0.25 ± 0.08Cc	0.60 ± 0.08Bb	1.19 ± 0.13Aa
48	32.05	d-Cadinene	na	1454	1467	--	--	0.52 ± 0.13Aa	0.79 ± 0.13Aa
49	35	Cedrol	0.00013–0.001 [35]	1487	1597	--	--	1.68 ± 0.32Bb	4.24 ± 0.10Aa
		**Irregular terpenes**							
50	3.09	Methyl isobutyl ketone	0.1–5 [24]	*	*	--	0.73 ± 0.13	--	--
51	14.75	(E)-4,8-dimethyl-1,3,7-nonatriene	na	1049	*	--	0.37 ± 0.08Bb	--	1.00 ± 0.16Aa
52	28.15	α-Ionone	0.001–0.006 [24]	1312	1411	1.30 ± 0.31	--	--	--
53	28.44	Geranylacetone	0.06 [36]	1316	1431	2.43 ± 0.52Aa	0.36 ± 0.08Bb	0.38 ± 0.08Bb	0.31 ± 0.03Bb
54	30.53	trans-á-Ionone	0.001–0.006 [24]	1339	1466	0.38 ± 0.11	--	--	--
		**N-containing compounds**							
55	5.5	N-Benzylaniline	na	1054	*	0.78 ± 0.16	--	--	--
56	22.41	Indole	0.5 [24]	1416	1307	--	1.15 ± 0.19	--	--
57	27.08	4-Pyrrolidinopyridine	na	1327	*	5.51 ± 0.54Bb	4.26 ± 0.4Cc	13.19 ± 0.49Aa	2.6 ± 0.43Dd
58	27.2	5-methyl-1,3-dihydro-2H-benzimidazol-2-one	na	2021	*	23.76 ± 1.37Aa	16.41 ± 0.5Bb	--	--
		**S-containing compounds**							
59	40.47	Cocarboxylase	na	1445	*	0.27 ± 0.07Aa	0.44 ± 0.02Aa	0.16 ± 0.06Aa	0.22 ± 0.10Aa
60	40.72	L-Methionine	750 [24]	*	*	0.44 ± 0.14	--	--	--

Note: ODT: odor detection threshold. All ODTs presented in this table constitute human olfactory thresholds in the air. “na” indicates that no ODT value was available in the references. -- indicates that the compound has not been detected. The different letters in each column represent significant differences at the 5% level. Different lower-case letters behind data indicate significant differences at *p* ≤ 0.05, and upper-case letters indicate significant differences at *p* ≤ 0.01 between taxa by using Duncan’s test. The compound data of each taxon was expressed as the mean ± SD of three samples. LRI (calc), linear temperature-programmed retention indices calculated; LRI (lit), linear temperature-programmed retention indices reported in the literature [2,3,4,5,37] (LRI Database on the web: http://www.odour.org.uk/lriindex.html). * indicates that the retention index of the compound could not be calculated (the standard series of n-alkanes is not sufficient to calculate) or could not be retrieved.
